# Protective Role of Polydatin Against Vancomycin‐Induced Lung Toxicity via Oxidative Stress, Inflammation, Endoplasmic Reticulum Stress, Apoptosis, and Ferroptosis Pathways

**DOI:** 10.1002/jat.70203

**Published:** 2026-04-10

**Authors:** Serpil Aygörmez, Sefa Küçükler, Selim Çomaklı, Hasan Şimşek, Özge Kandemir, Fatih Mehmet Kandemir, Şaban Maraşlı

**Affiliations:** ^1^ Department of Biochemistry, Faculty of Veterinary Medicine Kafkas University Kars Türkiye; ^2^ Department of Biochemistry, Faculty of Veterinary Medicine Atatürk University Erzurum Türkiye; ^3^ Department of Pathology, Faculty of Veterinary Medicine Atatürk University Erzurum Türkiye; ^4^ Department of Physiology, Faculty of Medicine Aksaray University Aksaray Türkiye; ^5^ Department of Food Processing, Aksaray Technical Sciences Vocational School Aksaray University Aksaray Türkiye; ^6^ Department of Medical Biochemistry, Faculty of Medicine Aksaray University Aksaray Türkiye

**Keywords:** apoptosis, endoplasmic reticulum stress, ferroptosis, polydatin, vancomycin

## Abstract

This study aimed to evaluate whether polydatin (Poly) could eliminate the harmful effects of vancomycin (VCM) on the lungs of rats. Rats were administered VCM (200 mg/kg) and Poly (50 mg/kg), both separately and in combination, for a duration of 7 days. Following this, various methods were utilized to analyze proteins and pathological changes in lung tissue related to oxidative stress, inflammation, endoplasmic reticulum stress, apoptosis, and ferroptosis. It was found that Poly application significantly enhanced antioxidant enzyme activities and nonenzymatic antioxidants while decreasing VCM‐induced lipid peroxidation. The study showed that VCM elevated the expression of inflammatory cytokines as well as Janus kinase 2 (JAK‐2) and signal transducer and activator of transcription 3 (STAT‐3), whereas Poly treatment inhibited these genes. Furthermore, VCM was found to induce apoptotic markers, but Poly offered protection to lung tissue against the harmful effects of VCM by demonstrating an antiapoptotic effect. Additionally, the increased expression of prostaglandin endoperoxide synthase 2 (PTGS2) and transferrin receptor 1 (TFR1), along with the decreased expression of glutathione peroxidase 4 (GPX4) in lung tissue induced by VCM, showed improvement following Poly administration. VCM was observed to increase, while Poly decreased the expression of endoplasmic reticulum stress markers. Overall, the findings of this study suggest that Poly has therapeutic potential in combating VCM‐induced lung toxicity.

## Abstract 2

This study evaluated the protective effects of polydatin (Poly) against vancomycin (VCM)‐induced lung toxicity. Rats received VCM (200 mg/kg) and Poly (50 mg/kg), alone or combined, for 7 days. Poly enhanced antioxidant defenses, reduced lipid peroxidation, inhibited JAK‐2/STAT‐3–mediated inflammation, regulated apoptotic and ferroptotic markers, alleviated endoplasmic reticulum stress, and protected lung tissue from VCM‐induced damage.

## 1 Introduction

Vancomycin (VCM), a glycopeptide antibiotic, is widely used to treat methicillin‐resistant Gram‐positive bacteria (Joshi et al. 2017; Qu et al. 2019). It is generally accepted as an antibiotic used in the treatment of septicemia, peritonitis, bacterial endocarditis, pseudomembranous colitis, meningitis, and pneumonia (El‐Gendy et al. 2025; Ibrahim et al. 2025). In addition to its treatment, side effects such as neutropenia, hypotension, and ototoxicity are frequently reported (El Bohi et al. 2021; Cui et al. 2022). VCM, which prevents the cross‐linking of the peptidoglycan wall of the bacteria, activates enzymes that cause cell damage by breaking down the cell wall (Yahia et al. 2025). The mechanisms underlying VCM‐induced toxicity have not been fully elucidated, but oxidative stress and inflammation are thought to play a role (Kandemir et al. 2018; El‐Gendy et al. 2025). It also activates apoptosis‐related pathways by causing mitochondrial dysfunction, DNA damage, and caspase activation (El‐Gendy et al. 2025). Oxidative stress caused by the imbalance between antioxidants and free radicals plays an important role in various conditions (Uhuo et al. 2022; Ibrahim et al. 2025). Oxidative stress causes a decrease in antioxidant enzymes and activation of inflammatory pathways (Al‐Sroji et al. 2023). Reactive oxygen species have an important effect on the oxidative balance of the body. Antioxidants play a role in neutralizing reactive oxygen species (Ibrahim et al. 2025). For these reasons, the therapeutic activities of natural compounds are important in protecting against multiple organ damage (Çağlayan et al. 2019; Kucukler et al. 2020). Ferroptosis, a type of iron‐dependent programmed cell death, involves the rupture of the mitochondrial outer membrane and an increase in mitochondrial density. Its biochemical characteristics are determined by lipid peroxidation (Zeng et al. 2023). The endoplasmic reticulum and mitochondria are connected to each other at multiple points through structures known as mitochondria‐associated endoplasmic reticulum membranes. Endoplasmic reticulum stress is known as homeostasis imbalance. Endoplasmic reticulum stress is associated with many diseases (Li et al. 2022). Following the study of the relationship between apoptosis and endoplasmic reticulum stress, in recent years, research has focused on the relationship between endoplasmic reticulum stress and ferroptosis (Wang et al. 2024). Even though the toxicities caused by VCM have been documented, the underlying molecular mechanisms leading to VCM‐induced pulmonary toxicity are insufficiently understood. Among these insufficient molecular mechanisms, the role of endoplasmic reticulum stress and ferroptosis pathways has not been investigated. Furthermore, the role of polydatin (Poly), known for its antioxidant and anti‐inflammatory properties, in modulating endoplasmic reticulum stress and ferroptosis pathways in VCM‐induced pulmonary toxicity has not yet been elucidated.

Poly is a natural compound found in grapes, hop cones, peanuts, hop pellets and cocoa‐containing products (Ali et al. 2022). Poly, widely used as a food flavoring, has significant nutritional value and is widely consumed in various Asian societies (Dahran et al. 2025). Poly, a stilbenoid glycoside, has important properties such as regulating carbohydrate and lipid metabolism, anti‐inflammatory and antioxidant (Bheereddy et al. 2021; Liu et al. 2023). Studies have reported that Poly provides protection against oxidative stress caused by various toxicants in lung tissue (Mostafa et al. 2021; Ali et al. 2022).

Studies on VCM in recent years have shown that VCM plays a role in hepatotoxicity and nephrotoxicity. However, there are no comprehensive studies on lung toxicity after VCM exposure. The presented study aimed to investigate the effects of Poly on VCM‐induced lung toxicity, which causes organ toxicities as well as its therapeutic properties, by biochemical, molecular, and histopathological analyses.

## 2 Material and Methods

### 2.1 Chemicals

VCM (Vancotek, Koçak Pharmaceutical, Türkiye) and Poly (98%, Cas No.: 65914‐17‐2) were used. All other chemicals were supplied by Sigma.

### 2.2 Ethics Committee Approval

Ethics committee approval for the study was obtained from Necmettin Erbakan University KONUDAM Experimental Medicine Application and Research Center Directorate (Konya/Türkiye) with the decision number 2025‐074.

### 2.3 Groups and Procedures

Twenty‐eight *Wistar albino* rats, weighing 220–250 g and aged 10–12 weeks, were used in the study. The animals were kept in cages in a controlled room with a constant temperature of 24°C–25°C and a 12‐h light–dark cycle. They were provided with access to unlimited amounts of water and standard food. All animal experiments were performed at Necmettin Erbakan University KONUDAM Experimental Medicine Application and Research Center (Konya/Türkiye). The 200 mg/kg dose of VCM was chosen based on findings from previous studies indicating that it causes organ toxicities. Poly's 50 mg/kg dose has been shown to exhibit antioxidant and anti‐inflammatory effects in previous studies. Therefore, Poly has not shown any side effects and is reported to be a safe and effective dose. VCM at a dose of 200 mg/kg and Poly at a dose of 50 mg/kg were deemed suitable to evaluate the protective effects of Poly against VCM‐induced pulmonary toxicity. This provides a reliable experimental model. *Wistar albino* rats were randomly divided into 4 groups, each consisting of 7 rats.

**Control:** Saline was administered intraperitoneally (i.p.) once daily for 7 days.

**Poly:** 50 mg/kg Poly was administered orally once daily for 7 days (Tong et al. 2020).

**VCM:** 200 mg/kg VCM was administered i.p. once daily for 7 days (Çağlayan et al. 2019).

**VCM+Poly:** 200 mg/kg VCM was administered i.p. once daily for 7 days, followed 30 min later by 50 mg/kg Poly orally once daily for 7 days. The experimental approach was chosen to better reflect a clinically relevant scenario in which a protective or adjunct agent is introduced shortly after exposure to a toxic drug to mitigate developing tissue injury.

### 2.4 Tissue Collection

Twenty‐four hours after the last VCM and Poly administration, rats were decapitated under light sevoflurane anesthesia and lung tissues were removed. Some of the lung tissues were taken for biochemical analyses and stored at −80°C until analyses were performed. The other part was placed in 10% formaldehyde solution for histological analysis.

### 2.5 Lipid Peroxidation and Antioxidant Analyses in Lung Tissue

Lung tissue was homogenized in 1.15% potassium chloride solution in a homogenizer (TissueLyser II, Qiagen, Netherlands) to obtain homogenate. The protein content of lung tissue was evaluated using the methods of Lowry et al. (1951), malondialdehyde (MDA) level by Placer et al. (1966), glutathione (GSH) level by Sedlak and Lindsay (1968), catalase (CAT) activity by Aebi (1984), superoxide dismutase (SOD) activity by Sun et al. (1988), and glutathione peroxidase (GPx) activity by Lawrence and Burk (1976).

### 2.6 Quantitative Real‐Time Polymerase Chain Reaction (qRT‐PCR) Analysis of Lung Tissue

To perform gene expression analyses in lung tissues of all experimental groups, relative mRNA transcript levels of nuclear factor erythroid 2‐related factor 2 (Nrf‐2), heme oxygenase 1 (HO‐1), nuclear factor kappa B (NF‐κB), tumor necrosis factor‐α (TNF‐α), interleukin 1β (IL‐1β), Janus kinase 2 (JAK‐2), signal transducer and activator of transcription 3 (STAT‐3), protein kinase R‐like ER kinase (PERK), activating transcription factor 6 (ATF‐6), serine/threonine‐protein kinase/endoribonuclease inositol‐requiring enzyme 1α (IRE1α), prostaglandin endoperoxide synthase 2 (PTGS2), glutathione peroxidase 4 (GPX4) and transferrin receptor 1 (TFR1) genes were analyzed by qRT‐PCR. QIAzol Lysis Reagent (Cat. No./ID: 79306) was used for this process. After the tissues were homogenized with 1 mL of QIAzol Lysis Reagent, 200 μL of chloroform was added, followed by vortexing and centrifugation at 15.000 rpm. After centrifugation, 3 phases were formed and the top transparent phase was transferred to new tubes and isopropanol was added. The tubes were then centrifuged again and the isopropanol was removed from the tubes. The pellet remaining at the bottom of the tube was washed with ethyl alcohol. After removing the ethyl alcohol from the tubes, the RNA sediment was dissolved with DNase/RNase‐free water, and its concentrations were determined using a NanoDrop (BioTek Epoch) device. In the second stage, cDNAs were synthesized from total RNAs with the iScript cDNA Synthesis Kit (1708891, Bio‐Rad, USA) and the third stage was started. In this final step, the reaction was set up with the primers whose sequences are given in Table 1, DNase/RNase‐free water and iTaq Universal SYBR Green Supermix (1725121, Bio‐Rad, USA) according to the manufacturer's instructions. The reaction was carried out on the Rotor‐Gene Q (Qiagen) instrument and CT values were obtained after the temperature and time cycles determined for the reaction were completed on the instrument. From these CT values obtained, relative mRNA transcript levels were calculated by the 2^−ΔΔCT^ method (Livak and Schmittgen 2001). β‐Actin was used as a housekeeping gene.

**TABLE 1:** Primer sequences.

Gene	Forward sequence (5′–3′)	Reverse sequence (3′–5′)
Nrf‐2	TTTGTAGATGACCATGAGTCGC	TCCTGCCAAACTTGCTCCAT
HO‐1	ATGTCCCAGGATTTGTCCGA	ATGGTACAAGGAGGCCATCA
NF‐κB	AGTCCCGCCCCTTCTAAAAC	CAATGGCCTCTGTGTAGCCC
TNF‐α	CTCGAGTGACAAGCCCGTAG	ATCTGCTGGTACCACCAGTT
IL‐1β	ATGGCAACTGTCCCTGAACT	AGTGACACTGCCTTCCTGAA
JAK2	TAGGTACGGAGTATCTCGTG	TGGAGTTATAGACAGCCAGG
STAT‐3	TACCTGGAGCAGCTTCATCA	GATCTCGCCCAAGAGGTTAT
PERK	GATGCCGAGAATCATGGGAA	AGATTCGAGAAGGGACTCCA
ATF‐6	TCAACTCAGCACGTTCCTGA	GACCAGTGACAGGCTTCTCT
IRE1α	GCAGTTCCAGTACATTGCCATTG	CAGGTCTCTGTGAACAATGTTGA
PTGS2	CTCAGCCATGCAGCAAATCC	GGGTGGGCTTCAGCAGTAAT
GPX4	TCTGAGCCGCTTATTGAAGCC	CACACGCAACCCCTGTACTT
TFR1	CCGGCCTATATGCTTGGGTA	CAAGGGAGCACTCTGAAGCA
β‐Actin	CAGCCTTCCTTCTTGGGTATG	AGCTCAGTAACAGTCCGCCT

### 2.7 Western Blot Analysis of Lung Tissue

Western blot analysis was performed by extracting total protein from 50 mg of lung tissue using radioimmunoprecipitation assay (RIPA) buffer. Protein concentration was quantified using the Pierce BCA Protein Assay Kit (Rockford, IL, USA). To separate proteins, sodium dodecyl sulfate‐polyacrylamide gel electrophoresis (SDS‐PAGE) was conducted on a 12% gel, with 20 μg of protein loaded into each well. The proteins were then transferred onto a membrane using the Trans‐Blot Turbo Transfer System (Bio‐Rad). The membrane was incubated overnight at 4°C with the following primary antibodies: B‐cell lymphoma 2 (Bcl‐2) (Cat. No.: sc‐7382, 1:1000, Santa Cruz Biotechnology, USA) and Bcl‐2‐associated X protein (Bax) (Cat. No.: sc‐7480, 1:1000, Santa Cruz Biotechnology, USA). GAPDH (Cat. No.: sc‐32233, 1:1000, Santa Cruz Biotechnology, USA) was used as an internal control. Subsequently, the membrane was incubated with the relevant secondary antibody, goat anti‐mouse IgG (Cat. No.: sc‐525409, 1:5000, Santa Cruz Biotechnology) for 1 h at room temperature. Protein bands were visualized using Clarity Western ECL Substrate (Bio‐Rad). Imaging was performed using the ChemiDoc MP Imaging System (Bio‐Rad), and band intensity was analyzed with Image Lab software (version 4.1, Bio‐Rad).

### 2.8 Lung Histopathology

Following euthanasia, lung samples were collected from the rats and fixed in 10% buffered formalin for 72 h. Following fixation, the lungs were processed using a routine histopathological procedure and embedded in paraffin. Five‐micrometer‐thick sections were then prepared and routinely stained for hematoxylin–eosin. Architectural deterioration in the lung tissue was assessed using a light microscope. Randomization was applied in determining the experimental groups in this study. Tissue samples were randomly assigned to groups. To prevent observer bias in histopathological evaluations, a blinding method was applied.

### 2.9 Immunohistochemical Study

Five‐micrometer‐thick lung sections were deparaffinized in xylene and rehydrated in a graded alcohol series. The tissues were then carefully boiled with EDTA buffer (pH 9.0) to retrieve the antigens. To block endogenous peroxidase activity, the sections were incubated with 3% H_2_O_2_ solution for 20 min. Then, tissues were incubated overnight at 4°C with the following primary antibodies: 8‐hydroxy‐2′‐deoxyguanosine (8‐OHdG) (mouse monoclonal, sc‐66036, 1:100, Santa Cruz Biotechnology) and Caspase 3 (rabbit polyclonal, AF6311, 1:100, Affinity). Following incubation, the sections were treated with secondary antibody (ab236466, Mouse and Rabbit Specific HRP/DAB IHC Detection Kit, Abcam). The sections were treated with 3‐amino‐9‐ethylcarbazole (AEC) and diaminobenzidine (DAB) chromogen and washed with distilled water. Finally, the sections were counterstained with hematoxylin and prepared for microscopic evaluation. With the aid of ImageJ software, the positive staining in lung tissue was identified, and the positive staining area was measured. Two pathologists separately assessed the intensity of the immunohistochemical reactions.

### 2.10 Statistical Analysis

The statistical analysis results of the data obtained at the end of the study were analyzed using IBM SPSS 26.0 and GraphPad Prism 8.0.1 software. Data were analyzed using one‐way analysis of variance (ANOVA) followed by Tukey's post hoc test, the assumptions of normality and homogeneity of variance were verified using the Shapiro–Wilk test respectively, confirming the appropriateness of parametric analysis. Data are presented as mean ± standard error (SE). Results with *p*‐values less than 0.05 were considered statistically significant.

## 3 Results

### 3.1 Protective Effect of Poly Against VCM‐Induced Oxidative Stress in Lung Tissue

Figure 1 shows lung tissue oxidant (MDA) levels and antioxidant (GSH, SOD, GPx, CAT) values due to VCM and Poly applications to rats. When MDA levels were evaluated, it was found that VCM administration caused lipid peroxidation and caused serious damage to the lung tissue compared to the control and Poly groups (*p* < 0.05). In rats exposed to VCM, a significant decrease in GSH levels and SOD, GPx, CAT activities was observed due to the increase in free radicals (*p* < 0.05). However, it was observed that MDA levels decreased and SOD, GPx, CAT activities and GSH levels increased in both the VCM and Poly groups compared to the VCM group. According to the results, it was determined that Poly application was effective against oxidative stress caused by VCM.

**FIGURE 1:**
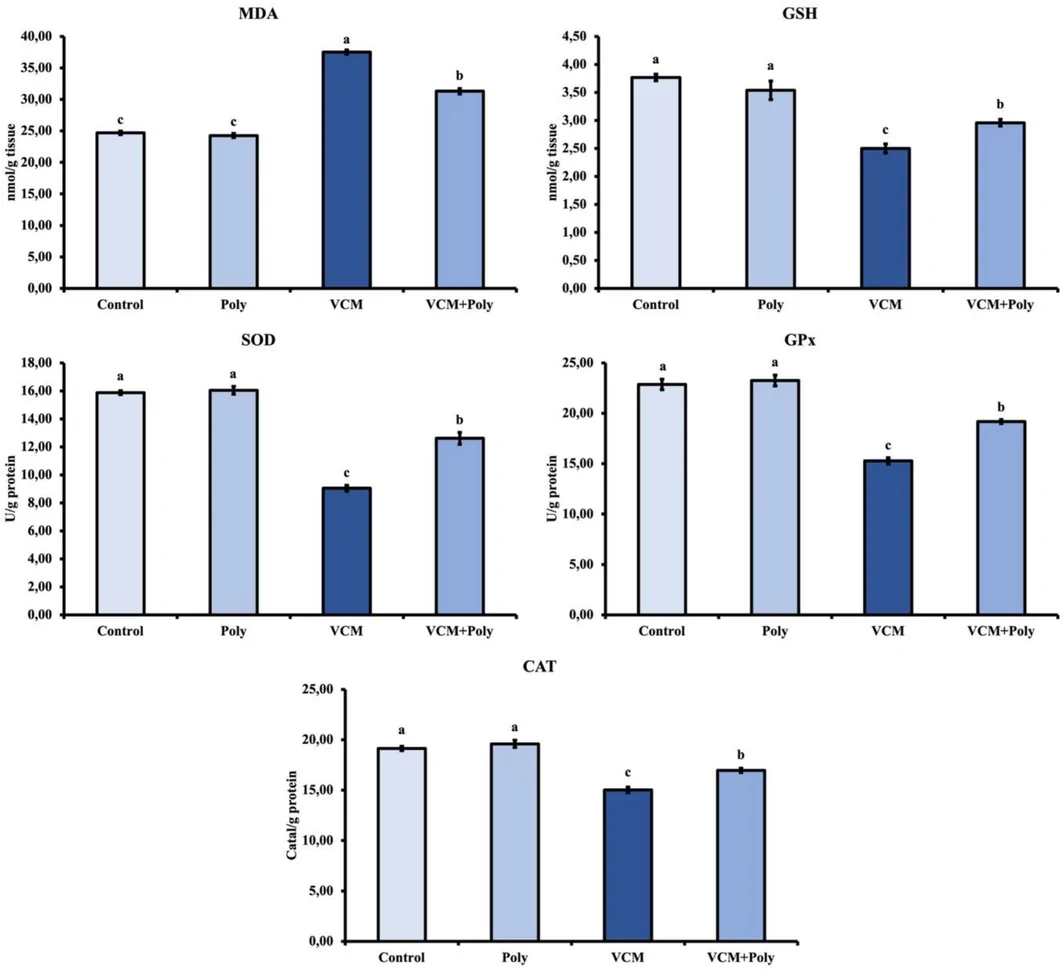
Effect of VCM and Poly administration on oxidative stress parameters in lung tissue. The values for each group are given as mean ± standard error (SE). ^a–c^: Different letters in the columns indicate statistical differences (*p* < 0.05).

### 3.2 Protective Effect of Poly on VCM‐Induced Nrf‐2 and HO‐1 Parameters in Lung Tissue

The mRNA transcription levels of Nrf‐2 and HO‐1, which have antioxidant properties, were analyzed in lung tissue (Figure 2). The mRNA transcription levels of Nrf‐2 and HO‐1 were found to be decreased in the VCM group (*p* < 0.05). In VCM + Poly, Nrf‐2 and HO‐1 parameters were found to be increased compared to the VCM group (*p* < 0.05).

**FIGURE 2:**
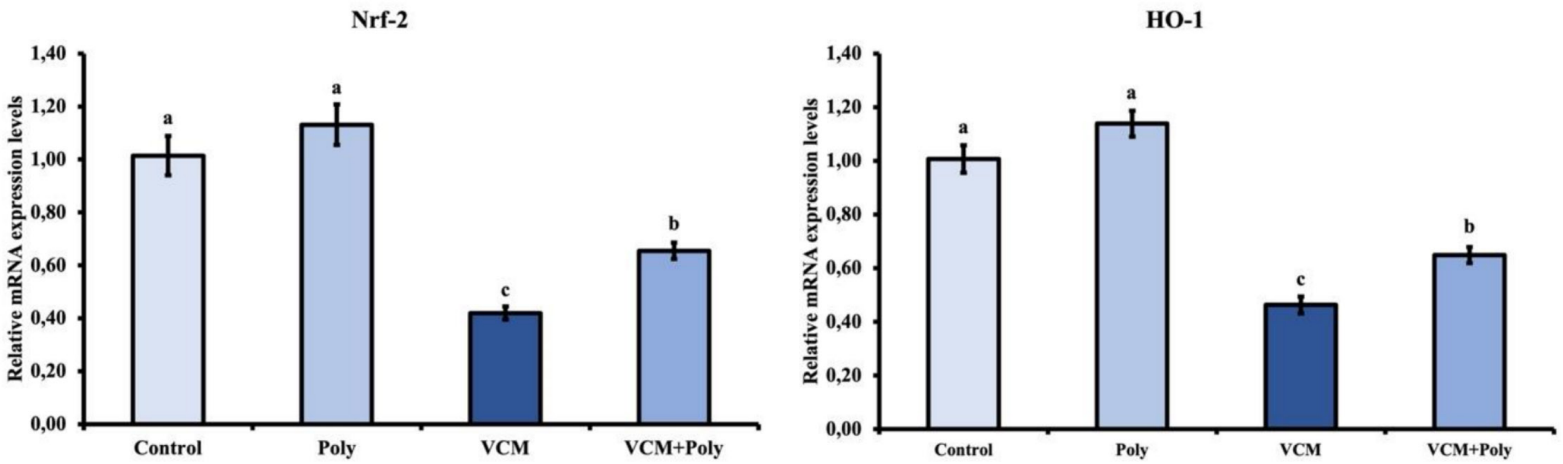
Effect of VCM and Poly administration on Nrf‐2 and HO‐1 parameters in lung tissue. Values for each group are given as mean ± standard error (SE). ^a–c^: Different letters in the columns indicate statistical differences (*p* < 0.05).

### 3.3 Protective Effect of Poly on VCM‐Induced Inflammation Parameters in Lung Tissue

In our study, mRNA expression levels of NF‐κB, TNF‐α, and IL‐1β genes, which regulate inflammatory responses and cytokine release, were determined by qRT‐PCR (Figure 3). Our findings showed that NF‐κB, TNF‐α, and IL‐1β gene expressions were significantly increased in the VCM group compared to the control group (*p* < 0.05). However, a significant decrease was detected in these parameters in the VCM + Poly group compared to the VCM group at the *p* < 0.05 level.

**FIGURE 3:**
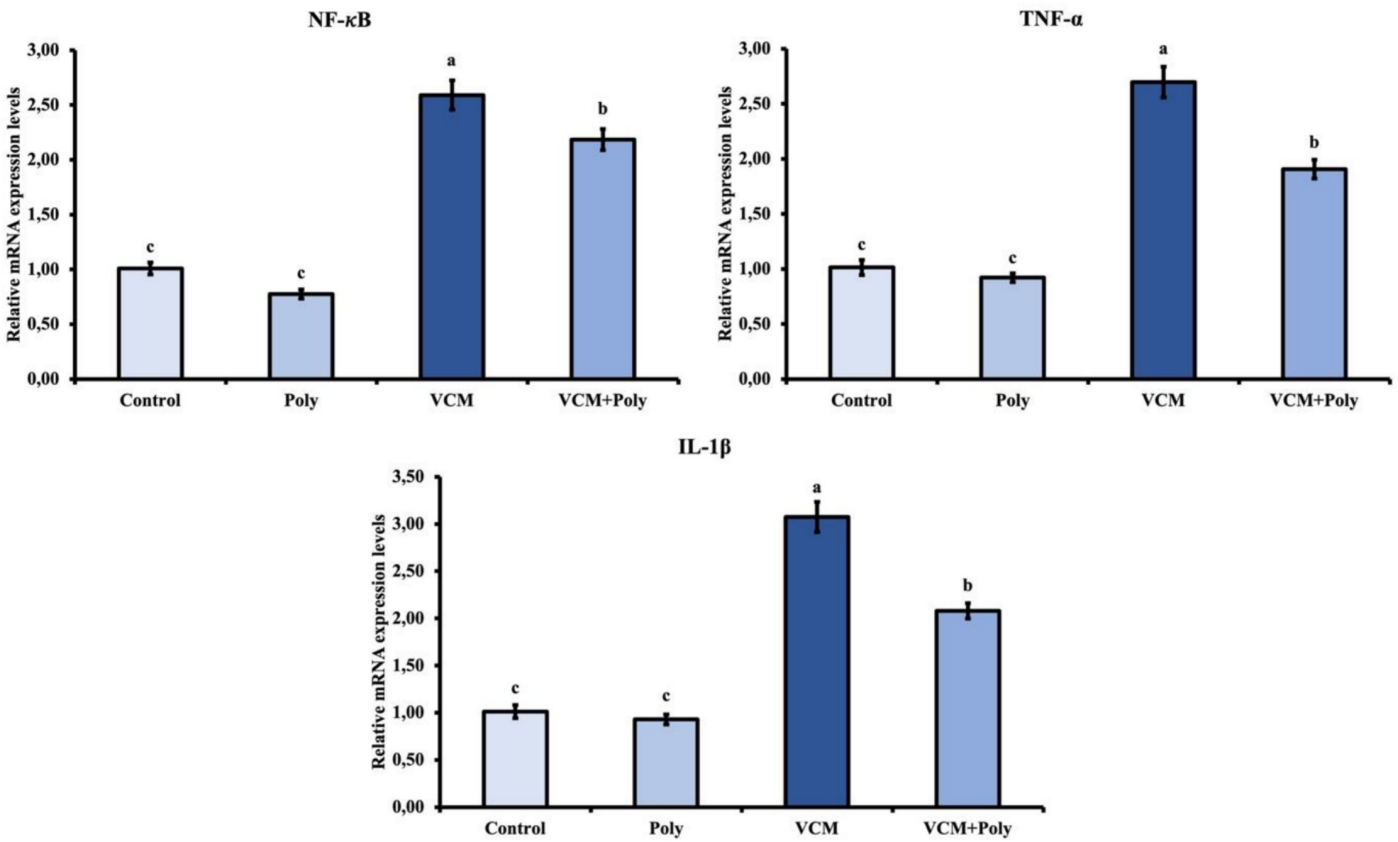
Effect of VCM and Poly administration on inflammation parameters in lung tissue. Values for each group are given as mean ± standard error (SE). ^a–c^: Different letters in the columns indicate statistical differences (*p* < 0.05).

### 3.4 Protective Effect of Poly on VCM‐Induced JAK‐2 and STAT‐3 Parameters in Lung Tissue

When the VCM group was compared with the control group, a significant increase was seen in the mRNA expression levels of both JAK‐2 and STAT‐3 (*p* < 0.05). No significant change was found in the Poly‐only group compared to the control group. Additionally, JAK‐2 and STAT‐3 mRNA expression levels were found to be significantly lower in both VCM and Poly treatments compared to the VCM group (Figure 4).

**FIGURE 4:**
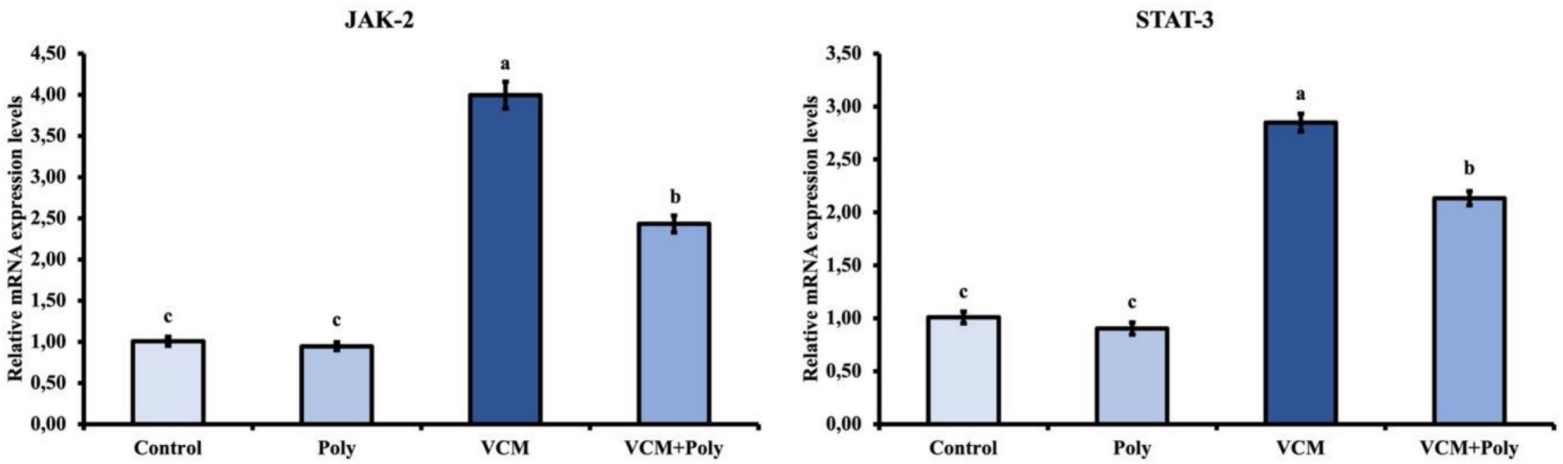
Effect of VCM and Poly administration on JAK‐2 and STAT‐3 parameters in lung tissue. Values for each group are given as mean ± standard error (SE). ^a–c^: Different letters in the columns indicate statistical differences (*p* < 0.05).

### 3.5 Protective Effect of Poly on VCM‐Induced Endoplasmic Reticulum Stress Parameters in Lung Tissue

Figure 5 shows the PERK, ATF‐6, IRE1α mRNA expression values in lung tissue due to VCM and Poly applications to rats. When the VCM group was compared with the control group, endoplasmic reticulum stress markers (PERK, ATF‐6, IRE1α) were significantly higher in the VCM group (*p* < 0.05). When VCM and Poly were applied together, decreases in PERK, ATF‐6, IRE1α mRNA transcription levels were detected (*p* < 0.05).

**FIGURE 5:**
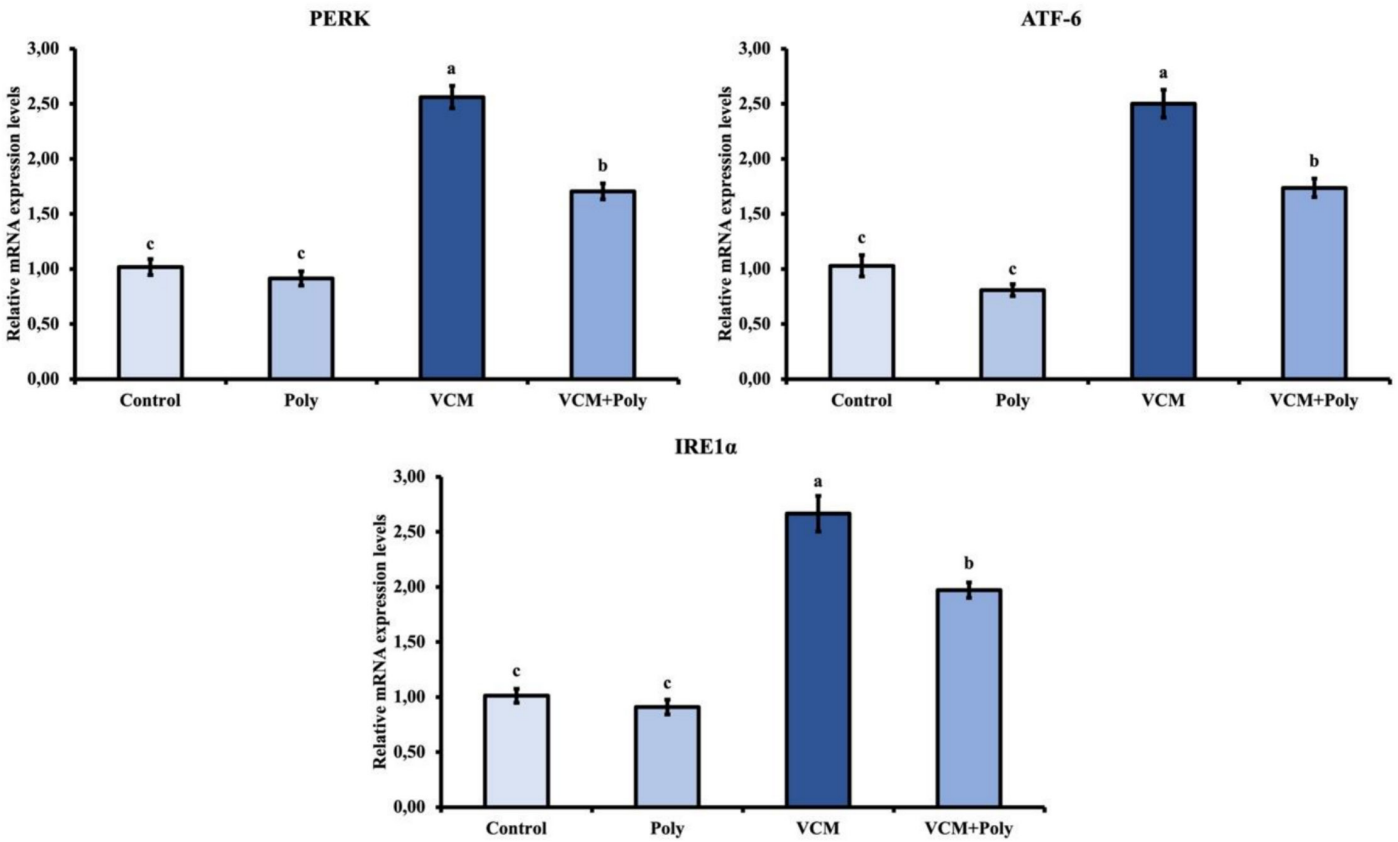
Effect of VCM and Poly application on endoplasmic reticulum stress parameters in lung tissue. Values for each group are given as mean ± standard error (SE). ^a–c^: Different letters in the columns indicate statistical differences (*p* < 0.05).

### 3.6 Protective Effect of Poly on VCM‐Induced Ferroptosis Parameters in Lung Tissue

The mRNA expression levels of ferroptosis markers (PTGS2, GPX4, TFR1) in the lung tissue of rats treated with VCM and Poly are shown in Figure 6. VCM exposure increased PTGS2 and TFR1 mRNA expression levels while decreasing GPX4 mRNA expression level compared to the control group (*p* < 0.05). Both VCM and Poly application were found to decrease PTGS2 and TFR1 mRNA expression levels compared to the VCM group while increasing GPX4 mRNA expression level.

**FIGURE 6:**
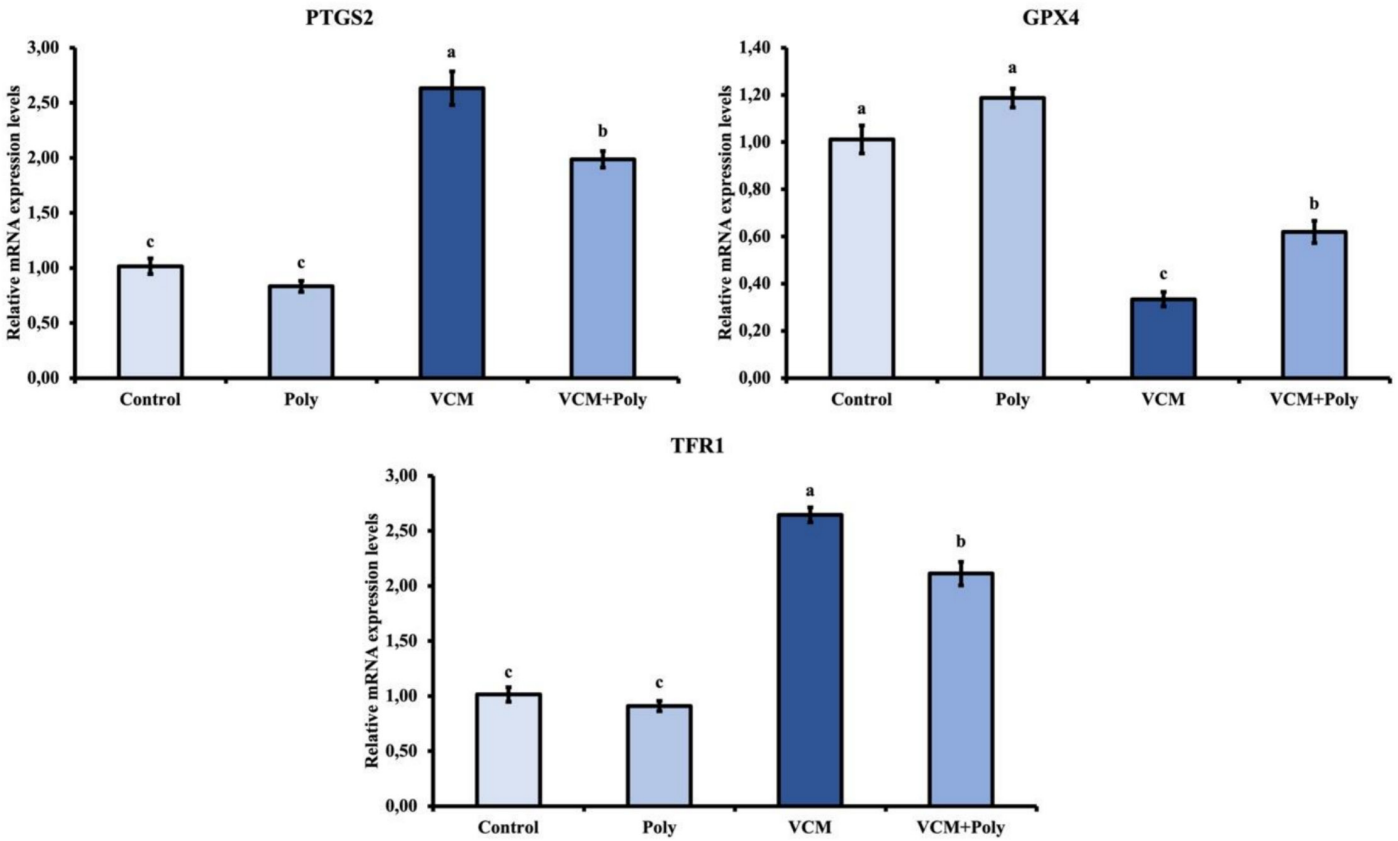
Effect of VCM and Poly application on ferroptosis parameters in lung tissue. Values for each group are given as mean ± standard error (SE). ^a–c^: Different letters in the columns indicate statistical differences (*p* < 0.05).

### 3.7 Protective Effect of Poly on VCM‐Induced Apoptosis Parameters in Lung Tissue

Bax and Bcl‐2 protein levels in lung tissue using western blot method are presented in Figure 7. According to the data, Bax protein level was up‐regulated and Bcl‐2 protein level was down‐regulated as a result of VCM application. In the VCM + Poly group, Bax protein level was suppressed and Bcl‐2 expression was induced.

**FIGURE 7:**
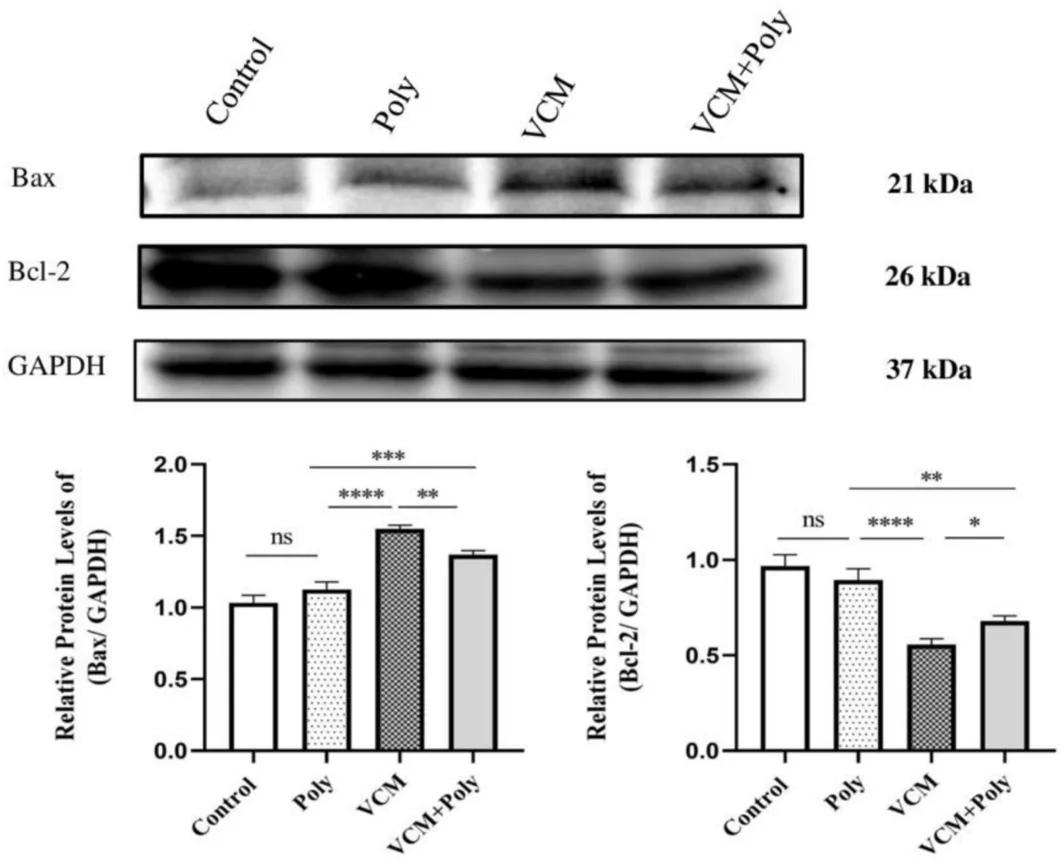
Effects of VCM and Poly administration on Bax and Bcl‐2 protein levels in lung tissue. Values for each group are given as mean ± standard error (SE). *p < 0.05, **p < 0.01, ***p < 0.001, ****p < 0.0001.

### 3.8 Light Microscope Results

H&E‐stained lung sections from control (Figure 8a) and Poly‐treated (Figure 8b) rats showed normal lung architecture. Thin‐walled alveoli and alveolar sacs were clearly visible. Marked histopathological changes were observed in lung sections from the VCM‐treated rats. Significant thickening of the interalveolar septa was noted, and narrowed alveoli and vascular hyperemia were also observed. Mononuclear cell infiltration was evident in the parenchymal regions, and there was a marked increase in the number of macrophages. The bronchiolar epithelium was vacuolized, and sloughed epithelial cells (Figure 8c). On the other hand, lung architecture was significantly improved in the VCM plus Poly group. The alveolar ducts were almost structurally preserved. The thickening of the interalveolar septa was decreased (Figure 8d).

**FIGURE 8:**
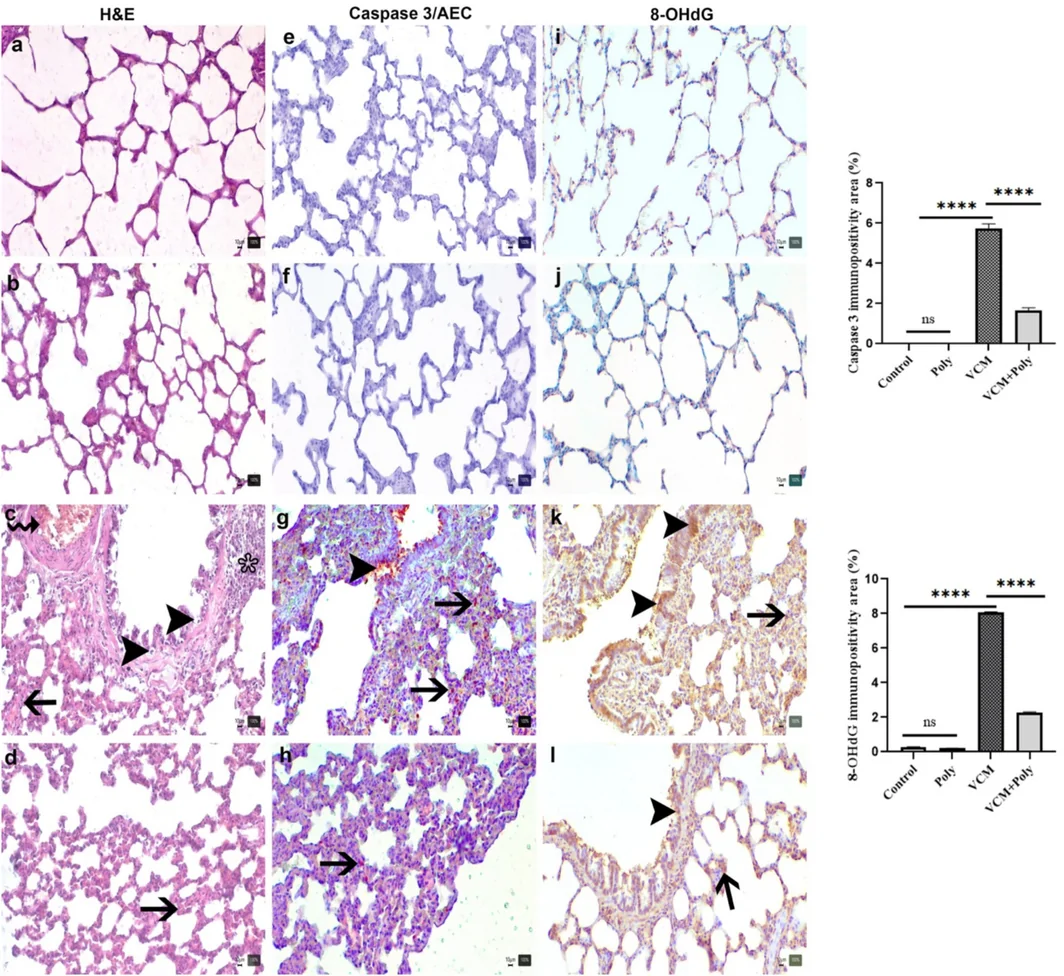
Photomicrographs of H&E‐stained, 8‐OHdG, and Caspase 3 immunopositivity sections of lungs of rats from different groups, showing the following: Control (a) and Poly‐treated (b) groups: Apparently normal alveolar duct, alveoli, and inter‐alveolar septa. VCM‐treated group (c): Thick interalveolar septa (arrow), peribronchiolar cell infiltration (asterisk), hyperemia in blood vessel (zigzag arrow), vacuolization in bronchiolar epithelium (arrowheads). VCM + Poly‐treated group (d): Mild thick interalveolar septa (arrow). Control (e) and Poly‐treated (f) groups: negative Caspase 3 immunoreaction in the epithelial cells lining the alveoli. VCM‐treated group (g): intense immunoreactivity in the bronchiolar epithelium (arrowhead) and inflammatory cells (arrows). VCM + Poly‐treated group (h): few immunopositive cells in inflammatory cells (arrow). Control (i) and Poly‐treated (j) group: negative 8‐OHdG immunoreaction in the epithelial cells lining the alveoli. VCM‐treated (k): numerous cells (arrow: inflammatory cells, arrowheads: bronchiolar epithelium) with a strong 8‐OHdG immunoreaction. VCM + Poly‐treated group (l): few cells (arrow: inflammatory cells, arrowhead: bronchiolar epithelium) with a faint immunoreaction. Data are expressed as mean ± SE (*n* = 7). ****p < 0.05 compared with the control and VCM group.

### 3.9 Immunohistochemical Results

Examination of Caspase 3 immunostained lung sections from the control (Figure 8e) and Poly‐treated (Figure 8f) groups did not show Caspase 3 immunoreactivity (*p* > 0.05). In contrast, sections from the VCM‐treated group showed a strong Caspase 3 immunoreactivity in cells of interalveolar areas and bronchiolar epithelium (Figure 8g, *p* < 0.0001). On the other hand, sections from the VCM plus Poly‐treated group showed a weak Caspase 3 immunoreactivity in only a few cells (Figure 8h, *p* < 0.0001).

Immunohistochemical examination of lung tissue sections obtained from the control (Figure 8i) and Poly‐treated (Figure 8j) groups revealed negative 8‐OHdG immunoreactions (*p* > 0.05). In the VCM‐treated group, strong positive reactions were observed in the bronchial epithelial lining and the interstitial cell infiltrate (Figure 8k, *p* < 0.0001). Conversely, 8‐OHdG immunoreactions were significantly reduced in the VCM plus Poly‐treated group (Figure 8l *p* < 0.0001).

## 4 Discussion

VCM, which is utilized to combat gram‐positive bacteria, is recognized for causing various tissue toxicities. These toxic effects are linked to the activation of damage pathways due to oxidative stress. Therefore, this study aimed to investigate the impact of Poly, a natural food flavoring, on lung toxicity induced by VCM.

Due to a reduction or loss of antioxidant activity in cells, the organism becomes susceptible to oxidative stress, which in turn causes an increase in oxidants (Aksu et al. 2021; Ileriturk et al. 2022; Aygörmez, Makav, Kuru, Eliş Yıldız, et al. 2025). Reactive oxygen species, produced as normal products of cellular metabolism, play an important role in maintaining cellular signal transmission and homeostasis (Erişir et al. 2009; Aktas et al. 2017; Ozyigit et al. 2024; Aygörmez, Makav, Kuru, et al. 2025). Increased reactive oxygen species cause tissue damage as a result of the breakdown of cellular macromolecules (Aydin et al. 2009; Ileriturk and Kandemir 2023; Pan et al. 2023). Oxidative stress, which is the most important factor underlying the lung toxicity process, contributes to the development of oxidative damage as a result of the production of strong oxidants by increasing the production and secretion of inflammatory factors (Zhou et al. 2021; Varışlı et al. 2022). In the presented study, it was found that MDA levels increased, Nrf‐2 and GSH levels decreased, and CAT, GPx, HO‐1, and SOD enzyme activities decreased in the lung tissue of rats due to VCM‐induced toxicity. As a result, it was determined that oxidative stress occurred and that the resulting oxidative stress caused damage to the lung tissue. Studies on the subject have reported that VCM causes the accumulation of reactive oxygen species in tissues and cells, that increased reactive oxygen species disrupt the body's antioxidant system, leading to oxidative stress damage, and that it causes tissue damage by damaging the structure and functions of proteins (El‐Shoura et al. 2024; Ibrahim et al. 2025). It was found that the administration of Poly, a natural compound, together with VCM reduced the increased MDA level, decreased Nrf‐2 and GSH levels, and increased CAT, GPx, HO‐1, and SOD activities. It has been stated that Poly has the potential to suppress lipid peroxidation and oxidative stress and increase antioxidant activity against different toxic substances (Dahran et al. 2025; Zarneshan et al. 2025).

Inflammation is another significant mechanism in lung toxicity, and it is known that oxidative stress triggers inflammation. In case of oxidative stress, inflammatory cytokines are released by lung cells (Yesildag et al. 2022; Zhang et al. 2022). NF‐κB, found in the cytoplasm of normal cells, plays an important role in the inflammatory response in damage‐ and pathogen‐related conditions (Çelik et al. 2020; Aydın et al. 2024; Zhu et al. 2024). IL‐1β and TNF‐α, which are associated with the development of inflammatory diseases, are released in high amounts in lung disease and damage the lung tissue (Wang et al. 2022). STAT‐3 is a cytoplasmic protein that can be induced by growth factors and various cytokines. Activated STAT‐3 enters the nucleus and binds to the regulatory region of the target gene, regulating the expression of nucleotide sequences of genes such as TNF‐α (Li et al. 2021). The binding of growth factors and cytokines to membrane receptors causes JAK‐2 activation. Activated JAK‐2 induces STAT‐3 phosphorylation (Xie et al. 2025). The JAK‐2/STAT‐3 pathway, which is important in the pathogenesis of lung injury, is effective in biological processes such as apoptosis and inflammation and affects the alveolar‐capillary barrier integrity (Qi et al. 2024; Xu et al. 2025). In the presented study, it was determined that VCM increased the mRNA expression levels of NF‐κB, JAK‐2, IL‐1β, STAT‐3, and TNF‐α in the lung tissue of rats, resulting in inflammation, and the resulting inflammation caused damage to the lung tissue. Studies have reported that the inflammatory response, characterized by the infiltration of inflammatory cells in rats administered VCM, causes exacerbation of tissue damage and disruption of tissue integrity as a result of oxidative stress caused by increased reactive oxygen species (Mehanna et al. 2022; Alrshedi et al. 2026). In the presented study, it was found that both VCM and Poly administration decreased the increased mRNA expression levels of NF‐κB, JAK‐2, IL‐1β, STAT‐3, and TNF‐α. In studies on the subject, it has been reported that Poly improves pulmonary dysfunction by preventing the alveolar‐capillary barrier, fibrosis, and alveolar epithelial cell damage in the inflammatory response against different chemical toxic substances, prevents apoptotic pathways in the lung tissue, and as a result protects tissue integrity (Li et al. 2014; Ali et al. 2022).

The endoplasmic reticulum is the organelle responsible for sorting and folding proteins (Baek et al. 2020; Varışlı et al. 2023). In case of oxidative stress, endoplasmic reticulum function is impaired and leads to the accumulation of unfolded/misfolded proteins, causing endoplasmic reticulum stress (Baek et al. 2020; Caglayan et al. 2022). Unfolded proteins play a role in the pathogenesis of various diseases (Yang et al. 2021). The accumulation of reactive oxygen species in the endoplasmic reticulum, which is the organelle most sensitive to reactive oxygen species, triggers endoplasmic reticulum stress in the progression and pathogenesis of lung injury (Zeng et al. 2023; Weng et al. 2023). Endoplasmic reticulum stress caused by reactive oxygen species triggers cellular ferroptosis (Dong et al. 2023; Huang et al. 2025). Endoplasmic reticulum stress participates in the ferroptosis process via IRE1α (Li et al. 2022). Ferroptosis is accelerated by cellular iron loading and results from lipid peroxidation (Wu et al. 2024). The newly discovered cell death, ferroptosis, differs from apoptosis in that it is structurally controlled by GPX4 (Wang et al. 2024; Wu et al. 2024). Additionally, ferroptosis plays an important role in the pathogenesis of lung injury (Li et al. 2022). In the presented study, it was found that VCM application increased the mRNA expression levels of PERK, ATF‐6, IRE1α, which are markers of endoplasmic reticulum stress, and PTGS2 and TFR1, which are ferroptosis markers, and decreased GPX4, which is also a ferroptosis marker. As a result, increased reactive oxygen species in the lung tissue triggered endoplasmic reticulum stress, and the resulting endoplasmic reticulum stress caused ferroptosis. In different studies on the subject, it has been reported that antibiotics cause endoplasmic reticulum stress by increasing the accumulation of misfolded proteins as a result of increasing reactive oxygen species, and the resulting endoplasmic reticulum stress disrupts tissue integrity (Dai et al. 2014; Laorodphun et al. 2022). In a study, it was stated that different toxic substances cause endoplasmic reticulum stress and the resulting endoplasmic reticulum stress causes ferroptosis, and the resulting endoplasmic reticulum stress and ferroptosis cause lung damage (Li et al. 2022; Wang et al. 2024). In the presented study, it was determined that Poly treatment with VCM decreased the mRNA expression levels of PERK, ATF‐6, IRE1α, which are markers of increased endoplasmic reticulum stress, and PTGS2 and TFR1, which are ferroptosis markers, and increased the mRNA expression level of GPX4, which is also a ferroptosis marker. As a result, it preserved tissue integrity by improving endoplasmic reticulum stress and ferroptosis in the lung tissue. It has been reported that natural compounds with antioxidant properties prevent ferroptosis by alleviating endoplasmic reticulum stress and inhibiting lipid peroxidation by preventing the accumulation of misfolded proteins caused by the increase in reactive oxygen species (Chen et al. 2024).

Apoptosis occurs via death receptor and mitochondria‐mediated pathways. Caspases in cells, which are inactive before the apoptotic process, become active when the apoptotic process begins (Khalil et al. 2020; Kızıl et al. 2023; Yılmaz et al. 2024). Caspase 3, the mediator of apoptosis in cells, initiates the apoptotic process (Aksu et al. 2018; Mehanna et al. 2022). Mitochondrial membrane integrity is important in apoptosis, and Bcl‐2, located in the outer mitochondrial membrane, regulates mitochondrial membrane integrity. An intrinsic proapoptotic pathway, Bax, controls apoptosis (Khalil et al. 2020; Kandemir and Dalkılınç 2025). In the extracellular pathway, TNF‐α is an important inducer of cell apoptosis (Le et al. 2022). Activated proapoptotic protein Bax eliminates mitochondrial membrane integrity, releases apoptogenic mitochondrial proteins into the cytosol, and activates Caspase 3, leading to apoptosis. Bcl‐2 antagonizes the apoptosis process (Khalil et al. 2020; Le et al. 2022). Additionally, oxidative stress plays an important role in the pathogenesis of VCM‐induced tissue toxicity (El Bohi et al. 2021; Yu et al. 2022). In the presented study, as a result of western blot analysis of VCM, it was found that the amount of Bax protein increased, the amount of Bcl‐2 protein decreased, and as a result of Caspase 3 immunohistochemical analysis, it showed a strong Caspase 3 immunoreactivity in the interalveolar area and bronchiolar epithelial cells, and as a result of 8‐OHdG immunohistochemical analysis, strong positive reactions were detected in the bronchial epithelial lining and interstitial cell infiltration. Studies on the subject have stated that, in response to increased reactive oxygen species and inflammation, VCM activates the depolarization of the mitochondrial membrane potential and leads to apoptosis (Kucukler et al. 2020; Mehanna et al. 2022). In this study, it was found that both VCM and Poly treatment decreased the amount of Bax protein in the lung tissue of rats as a result of western blot analysis, increased the amount of Bcl‐2 protein, and Caspase 3 immunohistochemically showed a weak Caspase 3 immunoreactivity in only a few cells, and 8‐OHdG immune reactions were significantly reduced. Studies have reported that Poly improves apoptosis in rats due to its antioxidant and anti‐inflammatory effects in tissue toxicity against different toxic substances (Tong et al. 2020; Dahran et al. 2025).

### 4.1 Study Limitations

It should be noted that this study has some limitations. Although Poly showed protective effects against VCM‐induced lung toxicity, the parameters Nrf‐2, HO‐1, NF‐κB, TNF‐α, IL‐1β, JAK‐2, STAT‐3, PERK, ATF‐6, IRE1α, PTGS2, GPX4, and TFR1 were not evaluated at the protein level. Therefore, further research involving the evaluation of protein levels of Nrf‐2, HO‐1, NF‐κB, TNF‐α, IL‐1β, JAK‐2, STAT‐3, PERK, ATF‐6, IRE1α, PTGS2, GPX4, and TFR1 parameters is needed to provide a more comprehensive understanding of Poly's potential role and mechanism of action on pulmonary toxicity. In our current study, PTGS2, GPX4, and TFR1 mRNA expression levels were analyzed to demonstrate the role of ferroptosis. However, another limitation of this study is that while it provides evidence supporting ferroptosis and these parameters are used in publications, full validation requires additional ferroptosis‐specific indicators such as ACSL4, lipid reactive oxygen species, and iron levels, which are key regulators. The absence of assessment of key regulators such as ACSL4, lipid reactive oxygen species, and iron levels represents a limitation of our study. Therefore, although our current findings suggest a potential role for ferroptosis in mitigating VCM‐induced pulmonary toxicity by Poly, further studies involving comprehensive analyses are needed to fully determine ferroptotic cell death. Future studies should address these limitations to further validate them.

## 5 Conclusion

These findings demonstrate that VCM can cause lung toxicity through various mechanisms, including oxidative stress, inflammation, endoplasmic reticulum stress, apoptosis, ferroptosis, and histopathological defects. Poly application was found to reduce oxidative stress caused by VCM and strengthen the antioxidant defense system. It also potentially prevents inflammation, endoplasmic reticulum stress, apoptosis, and ferroptosis. Additionally, it demonstrates the ability to reduce cell death processes by stabilizing the mitochondrial membrane. In conclusion, Poly application may be a novel and promising treatment for VCM‐induced lung toxicity.

## Author Contributions

All authors contributed to the study's conception and design. Serpil Aygörmez, Sefa Küçükler, Selim Çomaklı, Hasan Şimşek, Özge Kandemir, Fatih Mehmet Kandemir, Şaban Maraşlı performed material preparation, data collection, and analysis. Serpil Aygörmez wrote the first draft of the manuscript, and all authors commented on previous versions. All authors read and approved the final manuscript.

## Funding

The authors have nothing to report.

## Ethics Statement

All animal experiments complied with the ARRIVE guidelines (https://arriveguidelines.org). Ethics committee approval for the study was obtained from Necmettin Erbakan University KONUDAM Experimental Medicine Application and Research Center Directorate (Konya/Türkiye) with the decision number 2025‐074.

## Consent

The authors have nothing to report.

## Conflicts of Interest

The authors declare no conflicts of interest.

## Data Availability

Data will be made available on request.
